# Improving medical machine learning models with generative balancing for equity and excellence

**DOI:** 10.1038/s41746-025-01438-z

**Published:** 2025-02-14

**Authors:** Brandon Theodorou, Benjamin Danek, Venkat Tummala, Shivam Pankaj Kumar, Bradley Malin, Jimeng Sun

**Affiliations:** 1 University of Illinois at Urbana-Champaign, Urbana, IL USA; 2 Keiji AI, Seattle, USA; 3 Vanderbilt University, Nashville, TN USA; 4 Present Address: Vanderbilt University Medical Center, Nashville, USA

**Keywords:** Risk factors, Computer science

## Abstract

Applying machine learning to clinical outcome prediction is challenging due to imbalanced datasets and sensitive tasks that contain rare yet critical outcomes and where equitable treatment across diverse patient groups is essential. Despite attempts, biases in predictions persist, driven by disparities in representation and exacerbated by the scarcity of positive labels, perpetuating health inequities. This paper introduces FairPlay, a synthetic data generation approach leveraging large language models, to address these issues. FairPlay enhances algorithmic performance and reduces bias by creating realistic, anonymous synthetic patient data that improves representation and augments dataset patterns while preserving privacy. Through experiments on multiple datasets, we demonstrate that FairPlay boosts mortality prediction performance across diverse subgroups, achieving up to a 21% improvement in F1 Score without requiring additional data or altering downstream training pipelines. Furthermore, FairPlay consistently reduces subgroup performance gaps, as shown by universal improvements in performance and fairness metrics across four experimental setups.

## Introduction

Health inequities remain a persistent challenge in the medical field, manifesting in various ways such as access to care^1^, clinical trial cohort diversity^2^, and disparate treatment outcomes^3,4^. These inequities are influenced by the complex interplay of clinical factors with patient demographic characteristics like gender, race, and socioeconomic status. While medical institutions strive to provide equitable treatment, systemic issues and biases are challenging to avoid in the healthcare system, often leading to unequal care delivery and outcomes.

The increasing adoption of machine learning (ML) models in healthcare to assist and accelerate tasks such as reading medical images^5–7^, diagnosing patients^8,9^, and predicting patient outcomes like mortality^10^ and readmission^11^ creates the need for large, high-quality training datasets. In practice, developing such datasets is challenging due to the sensitive nature of health data^12^, the rareness of target conditions or outcomes^13,14^, and the manual labor required by medical specialists^12^. Furthermore, ML models exhibit sensitivity to distributional shifts in patient populations^15,16^, further exacerbating the practical challenges of utilizing ML models to aid in providing patient care. The effect of dataset scarcity manifests in poor generalizability^17^ and unbalanced model performance among patient groups^18–22^, potentially perpetuating disparities in healthcare for underrepresented populations. These problems are prevalent and problematic even in large, curated, and popular healthcare datasets^23^.

Various dataset augmentation approaches have been deployed to reduce disparate performance stemming from unbalanced datasets. Upsampling involves increasing the representation of underrepresented or minority groups within the training data by augmenting or replicating instances from these groups^24–26^. This includes using synthetic data as demonstrated by^26–28^. Conversely, downsampling reduces the overrepresentation of majority groups in the training data by randomly excluding instances, thereby achieving a smaller but more balanced representation of various patient populations^25,29,30^. It has furthermore been proposed to train separate models for different groups to allow each to focus on the intricacies of group patterns^31^. Finally, several fairness-based models aim to explicitly incorporate fairness considerations during the model development and training process to ensure equitable outcomes^32–37^.

However, each of these approaches falls into one of the following two failure modes by definition:

1. **Weakening the dataset**. Throwing away, repeating, or using a subset of data all limit the distribution of data provided to the model and prevent them from learning the optimal patterns present in the full dataset.
2. **Weakening the training process**. Even beyond requiring intrusion upon the existing training and inference pipeline, adding additional fairness objectives can impede overall learning by enforcing a trade-off between performance and fairness as the model must choose whether to learn the patterns that best match the historical results or which best equalize different group representations, which are misaligned in the presence of data bias^38,39^, all without improving the underlying model capability or performance.

These issues are especially problematic given the unique challenges within the healthcare domain, where many tasks and datasets, such as those regarding mortality and readmission prediction, are inherently imbalanced and sensitive. The outsized importance of predicting the rare, critical outcomes relative to the baseline results places importance on maintaining both the overall level of performance and the ability to innovate in terms of method. Given this requirement, any effective technique to improve fairness cannot do so at the expense of performance and should operate over the dataset rather than interfere with method training and prediction. As such, using distracting training objectives, throwing away data, or performing low-quality synthetic augmentation, especially for those who struggle with the high dimensionality common within medical data, are suboptimal trade-offs. Instead, we explore the possibility of simultaneously improving both performance and fairness while flexibly and effectively supporting any type of input data.

**Fig. 1:**
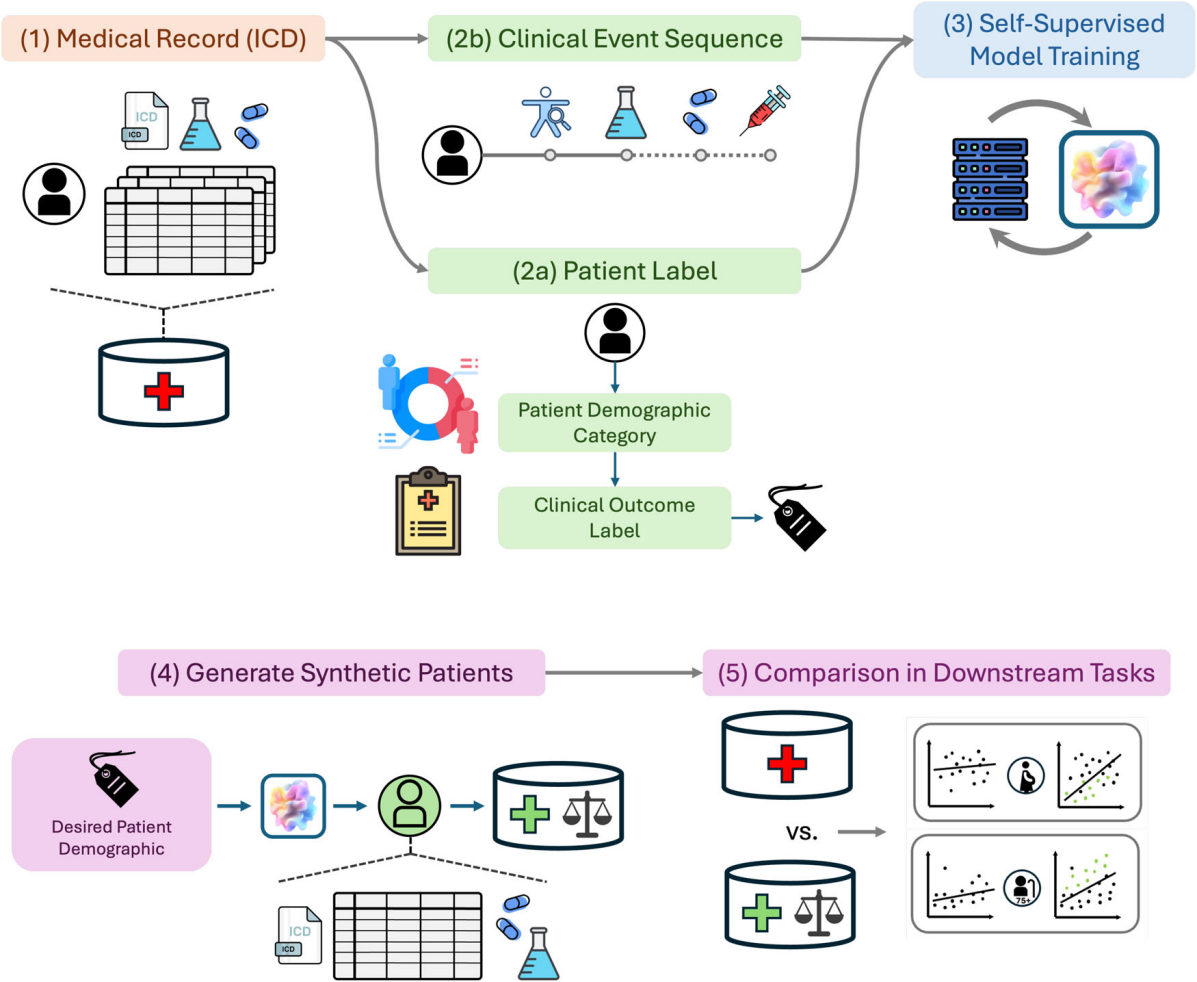
The FairPlay Pipeline: FairPlay leverages a conditional generative model to learn patterns within clinical datasets. The process begins by structuring source data into clinical event sequences, capturing patient interactions including diagnoses, lab tests, medications, and outcomes. Patient records are labeled according to demographic factors (insurance type, sex, race, age) and clinical outcomes (mortality, readmission). Patient labels, joined with patient event sequences are used to train the conditional generative model. After identifying inherent dataset imbalances and biases that could affect downstream model performance, FairPlay generates synthetic records for underrepresented groups and outcome classes. The resulting augmented dataset-combining original and synthetic records-is then used to train downstream machine learning models, leading to more equitable and accurate predictions.

To address these challenges, we propose to use large language model-based synthetic patient data, encapsulated in the approach FairPlay (shown in Fig. 1), to address the problem at its source. FairPlay offers a dual solution of enhancing overall algorithmic performance and mitigating bias by bolstering underrepresented populations. FairPlay generates realistic synthetic patient data, operating over the original, possibly high-dimensional dataset, to balance the underlying training data with respect to both outcome and any other characteristics to make it both fairer and easier to learn from, without limiting its quality or interfering with the training process or underlying model. FairPlayis enabled by the following technical contributions:

1. **Conditional and global pattern learning**. FairPlay uses a powerful probabilistic language model to learn the patterns of the underlying patient population both in aggregate and for specific populations and outcomes.
2. **Generation for dataset equalization**. FairPlay then samples from the learned distributions to conditionally generate new synthetic records of patients with desired characteristics. Those new records are added to the training dataset to balance the overall patient population and remove the incentive for bias.

We evaluate FairPlay across a variety of different patient populations and models on multiple datasets. We show that FairPlay can improve both the performance and fairness of downstream mortality prediction models. Specifically, we find that FairPlay can improve F1 Score averaged across a suite of downstream models by up to 21% without accessing any additional data or changing any of the downstream model pipelines, and achieves this for every subgroup while simultaneously shrinking the gap between different subgroup performances. This shrinking gap is demonstrated qualitatively and through the universal improvement of fairness metrics across four different experimental setups.

## Results

### Problem formulation

Our objective with our FairPlay approach is to offer a versatile solution that caters to various data types, tasks, and experimental designs. To create a foundation for this approach, we begin by delineating the healthcare ML problem and establishing clear notation for its components.

### Patient data

We represent a patient ${\mathcal{P}}$ as a triplet containing medical record data, protected characteristics, and outcome labels:

1 $${\mathcal{P}}=\left\{{\mathcal{R}},{\mathcal{C}},{\mathcal{L}}\right\}$$

The record, ${\mathcal{R}}$, encompasses the medical data that acts as input to ML algorithms. This data can be either static or sequential, and can comprise any mix of binary, categorical, and continuous data points, capturing medical variables such as patient diagnoses, medication regimens, lab tests, and more. These details may be aggregated into a single representation or ordered longitudinally over a series of patient visits across time. The protected characteristics, ${\mathcal{C}}$, are a set of attributes corresponding to group memberships, typically demographic, that we aim to monitor to ensure unbiased model output. Finally, the labels, ${\mathcal{L}}$, represent medical outcomes that may be predicted or classified by ML models, such as patient mortality or readmission.

### Vector representation

To allow input to ML models, we then convert ${\mathcal{R}}$ and ${\mathcal{L}}$ into standardized vector representations. Depending on the specific task and nature of input data, ${\mathcal{R}}$ might either become the matrix, **R**, in the case of sequential data or static vector **r**. Generally, we can interpret this transformation as producing a matrix, with the static vector acting as the special case of a matrix with a single column. Each column then reflects a visit, which is represented as a multi-hot vector with ones denoting the medical codes and other content present at that time step. We then transform ${\mathcal{L}}$ to either a single binary value *l* or a label vector **l**, which can be a one-hot or multi-hot based on the task, respectively. In this paper, we consider a variable-length matrix **R** and binary label variable *l*, which represent the positive or negative outcome for the entire patient sequence. However, it may also be applied just to the final time step with the rest having a negative label (as in the case of mortality prediction where the patient can be assumed to have survived all but possibly the final visit). Finally, ${\mathcal{C}}$ can be vectorized into either a one-hot or multi-hot vector depending on the number of protected characteristics under consideration.

### Patient classification and prediction

The overall goal for the general health ML task is the identification of a function *f*: **R** → *l* that maps input patient data to the corresponding classification label. This is achieved by training a ML model, ${\mathcal{M}}$, to approximate *f* by learning *P*(*l*∣**R**) based on the available training data. This trained model ${\mathcal{M}}$ is then evaluated on unseen testing data, $\hat{{\mathcal{D}}}$, achieving a quantifiable performance metric, *s*, such as accuracy or F1 Score. The primary goal is to find ${m}_{\theta }\in {\mathcal{M}}$ which maximizes the output of $S:{\bf{R}}\times {\mathcal{M}}\to s$, where $s\in {\mathbb{R}}$.

### Fair machine learning

However, it is crucial to note that each patient within the training and testing datasets is also associated with their respective protected characteristics, ${\mathcal{C}}$. So, in addition to attempting to maximize the overall performance metric, *s*, it is imperative for the learned mapping to be equitable with respect to ${\mathcal{C}}$. Specifically, we have a simultaneous goal to minimize the disparity in performance across the different groups:

2 $$\sum _{{d}_{i},{d}_{j}\in \hat{{\mathcal{D}}}}D(S({d}_{i}| {m}_{\theta }),S({d}_{j}| {m}_{\theta }))$$

Where $D:{\mathcal{S}}\times {\mathcal{S}}\to {\mathbb{R}}$ computes the difference in performance between two groups. In the context of this paper, we consider the absolute difference between performance scalars, but in principle, more complex difference metrics can be used. The subset of patients which have a particular characteristic, exhibit characteristic *c*_*i*_ are represented by ${d}_{i}\in \hat{{\mathcal{D}}}$. In the formulation, ${m}_{\theta }\in {\mathcal{M}}$ denotes an instantiation of the ML model, parameterized by weights *θ*.

### Experimental design

We design experiments to evaluate our proposed technique by answering the following questions.

- Does FairPlay’s label balancing and general augmentation boost overall performance?
- Can FairPlay improve the performance and reduce the disparities across different group categories?
- Is FairPlay capable of improving performance and reducing disparities across multiple types of characteristics at once?

### Datasets

In this study, we use a pair of publicly available inpatient datasets, PhysioNet eICU^40^ and PhysioNet MIMIC-IV (Medical Information Mart for Intensive Care IV)^41^.

Physionet eICU is a multi-center ICU database, with data from over 200,000 ICU stays across the country. MIMIC-IV is an electronic health records database containing de-identified health information for over 100,000 patients admitted to the intensive care units (ICUs) at the Beth Israel Deaconess Medical Center between 2008 and 2019. Both datasets encompass various clinical data, including demographics, diagnosis codes, procedures, medication administration records, laboratory measurements, and vital signs. These diverse data elements provide a comprehensive representation of the patient’s medical conditions and treatments during their ICU stays. The datasets are longitudinal in allowing multiple hospital visits for patients in the MIMIC dataset and multiple ICU stays within the eICU dataset, though the vast majority have only one.

For this study, we focus on specific data components, diagnosis medical codes, and lab values as the inputs to our downstream models. To prevent an explosion of tens of thousands of overly rare variables, we keep the fifty most common lab tests and aggregate the diagnosis codes up to the decimal point (so that I50.8, I50.9, and I50.92 all become I50). To provide the lab values in the same format alongside the binary diagnosis codes, we aggregate labs from throughout a hospital stay and bin them according to value deciles calculated for each unique lab. So, the medical variables can all be represented as binary inputs in a multi-hot vector for a given patient visit (Data representation details can be seen in Supplementary Fig. 2). We note that the MIMIC dataset is much more high-dimensional than the eICU dataset, with fixed-length vectors of size 9671 being fed to the downstream models compared to 1301 in eICU, allowing insight into such settings.

We then consider a variety of group memberships to analyze data bias and disparate model performance. Specifically, we explore two sets of protected characteristics per dataset, chosen based on the group statistics and the performance of baseline models to provide wide coverage of interesting experiments with clear group representation and performance disparities. We use age group and gender as protected characteristics for the eICU dataset and race and insurance type (as a proxy for income/socioeconomic status as in ref. ^23^) as protected characteristics for the MIMIC dataset. The statistics of the dataset can be found in Table 1, and information about the distributions of different group memberships is provided in later sections.

**Table 1:** Final Dataset Statistics

	eICU	MIMIC-IV
Number of Records	164,864	190,252
Mean Visits Per Record	1.189	2.318
Mean Variables Per Visit	87.114	54.727
Mortality Percentage	8.78%	15.5%
Unique Diagnoses	778	9145
Unique Labs	50	50
Total Unique Variables	1328	9695

### Task

For both datasets, we consider the exemplar healthcare ML task of in-hospital mortality prediction. It represents a sensitive and crucial task with a high degree of label skew (less than 16% positive labels in either dataset) and an outsized importance on correctly identifying positive labels. While both datasets are sequential, and we train FairPlay on a dataset of patients with possibly multiple visits, our downstream mortality prediction task is performed on a per-visit basis as would be done in a hospital setting. So, we avoid issues such as irregular numbers or gaps between visits as well as any requirement for censoring. To convert sequential labels into static ones, we assume that only the final visit has the potential for a positive label, with the rest remaining negative regardless of the patient’s final outcome.

### Metrics

Given the data’s inherent label skew and the importance of identifying positive instances, we use the F1 Score for model performance comparison:

3 $$F1=\frac{2\times \,\text{Precision}\times \text{Recall}}{\text{Precision}+\text{Recall}\,}$$

F1 Score prioritizes accurate high-risk patient identification while simultaneously rewarding false positive minimization.

Furthermore, in order to assess the disparity of the model performance across different groups, we have calculated a suite of fairness metrics. These metrics explore the comparative performance across majority and minority groups, which are ascertained according to the population demographics of the original training dataset.

We present what we feel are the two most comprehensive metrics, measuring the overall performance gap and spread in performance scores in our main paper, and we then include additional typical fairness metrics in Supplementary Table 4. The first of these metrics is *Disparate Impact*, which measures the ratio of F1 Scores between minority groups and the majority group baseline^42^. We calculate this metric for each minority group and present a weighted combination of the metric by the size of those groups. Here, the aim is to be as close to 1 as possible, signifying no difference in performance.

The second metric is *Theil Index*, which evaluates overall spread and inequality in F1 Scores across groups as a special case of the generalized entropy index^43^. Here, a lower value signifies a more even and fair spread in performances.

We then also include *Performance Variance*, *Equalized Odds*, *Equality of Opportunity*, and *Demographic Parity* in our supplement, and we provide further descriptions of those metrics there.

### Downstream models

Our FairPlay technique will be data, model, and pipeline agnostic. We aim to augment and debias the dataset without altering the predictive model training and deployment process.

So, we evaluate FairPlay and other data enhancement methods using standard ML techniques to assess data quality and bias. The different downstream ML methods are as follows:

- **k-Nearest Neighbors:** Locates similar training dataset patients to vote on a test patient’s label.
- **Logistic Regression:** Estimates label probability as a linear function of input variables.
- **Neural Network:** Models label probability through multiple hidden layers.
- **Random Forest:** Constructs decision trees to vote on the predicted label.
- **XGBoost:** An optimized gradient boosting method that predicts target variables using decision tree ensembles.

### Experimental setup

We deploy k-fold validation for comprehensive method testing and evaluation. For each dataset, we split the patients evenly between 5 folds. For each iteration, we apply FairPlay and our other compared data augmentation techniques to the patients within 4 of the folds. This creates new, enriched training datasets used to train each downstream model that are then evaluated on the remaining fold as our test dataset. We report our metrics averaged across the 5 folds along with their standard errors as a measure of variance, and we repeat this process with the same folds for each different experiment. We use the same hyperparameters (following^44^ and specified in the supplement), data format, and evaluation procedure for each fold and each experiment.

### Baseline methods

We compare FairPlay’s performance against the following established augmentation and fairness approaches:

1. **Original Dataset Only:** Uses the untouched training dataset for model training.
2. **Upsampling:** Duplicates minority label and group records to balance representation.
3. **Downsampling:** Removes majority label group records to balance representation.
4. **Separate Models:** Trains a separate prediction model for individual groups.
5. **SMOTE:** Synthetic Minority Over-sampling Technique generates synthetic samples in the feature space to balance class distribution^26^.
6. **MCRAGE:** Uses conditional tabular diffusion models to generate synthetic samples to balance class distribution^27^.
7. **SMA:** Uses autoregressive decision trees to generate a synthetic version of the original dataset, which is then upsampled to debias dataset class distributions^28^.

We want to note that the MCRAGE and SMA models were originally proposed and tested in different problem settings. Both explore extremely low-dimensional and largely non-binary datasets. Furthermore, SMA was tested with limited group distribution disparity. These models then struggle in a number of ways with our higher dimensional, more skewed, and binary data. First, the SMA autoregressive model fitting is inefficient, so while we produced results for the eICU dataset, the high-dimensional MIMIC dataset was computationally infeasible. Second, the SMA upsampling of the synthetic data is not problematic when the skew is small as there will be little to no repetition, but our more significant skew (and inclusion of outcome labels as “groups”) means that this approach yields a large amount of repetition and becomes in effect a weaker version of the original upsampling baseline. Third, the MCRAGE model’s diffusion approach is less readily adapted to our binary datasets. Finally, the overall performance of both models decays significantly in the high dimensional setting, especially with sparse variables. However, we want to make sure to note that these are not failings of the methods but differences in the experimental settings that we wanted to draw awareness to given the underperformance of these baselines on our experiments

### Overall performance

We begin by demonstrating the ability of FairPlay to improve the overall performance of downstream mortality prediction models through its synthetic data augmentation technique. For each dataset, we run our FairPlay pipeline without any protected characteristics, training a HALO model^44^ conditioned only on the mortality label. Subsequently, we generate deceased synthetic patients to balance the representation of mortality labels across patients in the augmented dataset. We then train a series of prediction models on the original dataset, this augmented dataset, and datasets modified by each applicable (not fairness-based) baseline approach. We compare these results in Table 2.

**Table 2:** Overall Mortality Prediction Performance

	Downstream Method F1 Score											
Training Data	eICU						MIMIC					
	Avg	KNN	LR	NN	RFC	XGB	Avg	KNN	LR	NN	RFC	XGB
Original Data	0.376 ± 0.02	0.180 ± 0.02	0.453 ± 0.03	0.486 ± 0.03	0.272 ± 0.02	0.490 ± 0.02	0.296 ± 0.00	0.112 ± 0.00	0.389 ± 0.00	0.428 ± 0.01	0.150 ± 0.00	0.400 ± 0.00
Upsampling	0.381 ± 0.01	0.249 ± 0.02	0.485 ± 0.01	0.427 ± 0.02	0.240 ± 0.02	0.504 ± 0.01	0.130 ± 0.00	0.113 ± 0.01	0.170 ± 0.01	0.202 ± 0.01	0.011 ± 0.00	0.155 ± 0.00
Downsampling	0.442 ± 0.01	0.273 ± 0.02	0.481 ± 0.01	0.481 ± 0.01	**0.487** ± **0.01**	0.486 ± 0.01	0.109 ± 0.00	0.053 ± 0.00	0.164 ± 0.00	0.150 ± 0.01	0.016 ± 0.00	0.161 ± 0.00
SMOTE	0.427 ± 0.01	0.289 ± 0.02	0.461 ± 0.01	0.446 ± 0.01	0.440 ± 0.02	0.500 ± 0.01	0.252 ± 0.00	**0.181** ± **0.01**	0.353 ± 0.00	0.210 ± 0.01	0.124 ± 0.00	0.393 ± 0.00
MCRAGE	0.376 ± 0.02	0.127 ± 0.01	0.454 ± 0.03	**0.513** ± **0.02**	0.295 ± 0.02	0.491 ± 0.03	0.298 ± 0.00	0.102 ± 0.00	0.389 ± 0.00	0.425 ± 0.00	0.172 ± 0.01	0.399 ± 0.00
SMA	0.280 ± 0.01	0.111 ± 0.01	0.379 ± 0.01	0.451 ± 0.02	0.091 ± 0.02	0.369 ± 0.02	–	–	–	–	–	–
FairPlay	**0.457** ± **0.01**	**0.292** ± **0.02**	**0.498** ± **0.01**	0.494 ± 0.01	**0.487** ± **0.01**	**0.513** ± **0.01**	**0.329** ± **0.00**	0.128 ± 0.00	**0.401** ± **0.01**	**0.442** ± **0.01**	**0.266** ± **0.00**	**0.407** ± **0.01**

Bold fonts indicates the best performing model.

The results indicate that FairPlay can enhance predictive performance, noticeably improving test F1 Score over baseline downstream models trained on the original, untouched dataset. Specifically, it improves the average F1 Score across all of the downstream models and folds by 21.5% from 0.376 to 0.457 in the eICU dataset and by 11.1% from 0.296 to 0.329 in the MIMIC dataset. This performance boost is achieved despite not accessing any additional data or changing any of the downstream model training methods. Instead, powerful auto-regressive language models are utilized to augment the dataset with its existing patterns.

Furthermore, the other approaches to augmenting or balancing the dataset are much less effective. This is especially true with the higher dimensional MIMIC dataset where each baseline except MCRAGE reduces performance on average (two of which do so by over 50%). However, even on the eICU dataset, none of the compared approaches improve performance as much as, or consistently across all downstream models as FairPlay. These results showcase FairPlay’s performance as the best method for general data-based performance enhancement.

### Group performance

**Fig. 2:**
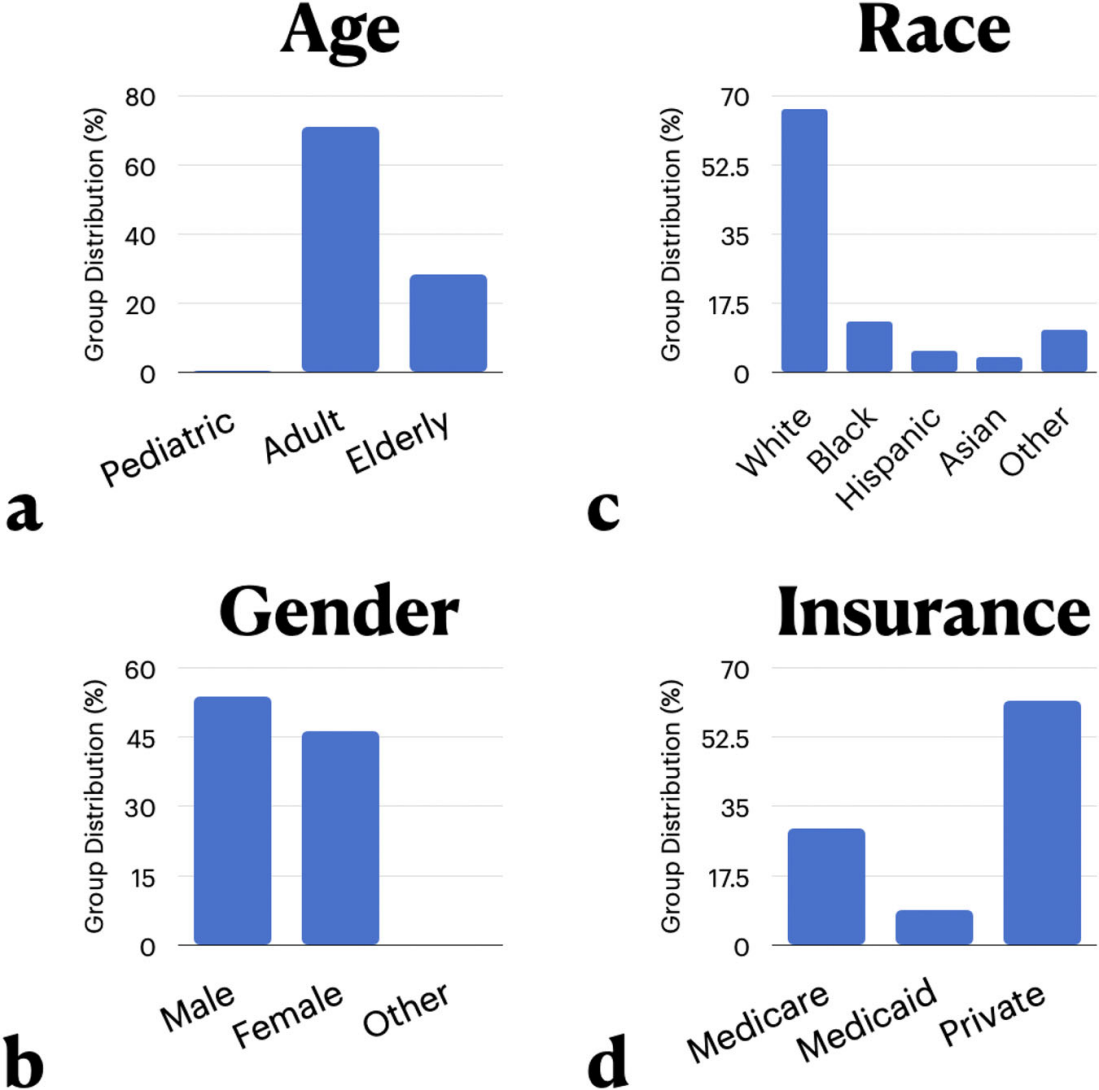
Group Distributions Across Experimental Attributes: the proportions of different subgroups within the different categories that define our four experiments. **a** shows the age subgroups with adults as the majority group and elderly and pediatrics as the minority groups (with pediatrics extremely small). **b** shows the race subgroups with whites as the majority group and the rest as minority groups. **c** shows the gender subgroups with males as the majority group and females as the minority groups (with others extremely small). **d** shows the insurance type subgroups with private as the majority group and Medicare and Medicaid as the minority groups.

Next, we explore the stratification of performance across different groups and evaluate whether FairPlay can improve not only both the overall performance but also the performance of each group to reduce performance disparities. To that end, we perform four different experiments, two on each dataset. For each experiment, we run FairPlay with the groups corresponding to a single attribute as protected characteristics, balancing the dataset over each different mortality label and group membership pair. Specifically, we operate over age and gender for the eICU dataset, and explore race and insurance type (as a proxy for socioeconomic status) for the MIMIC dataset. We provide the distributions of different groups for each experiment in Fig. 2, and enumerate exact group definitions (e.g., age cut-offs and specific to general racial group mappings) in our supplement.

**Fig. 3:**
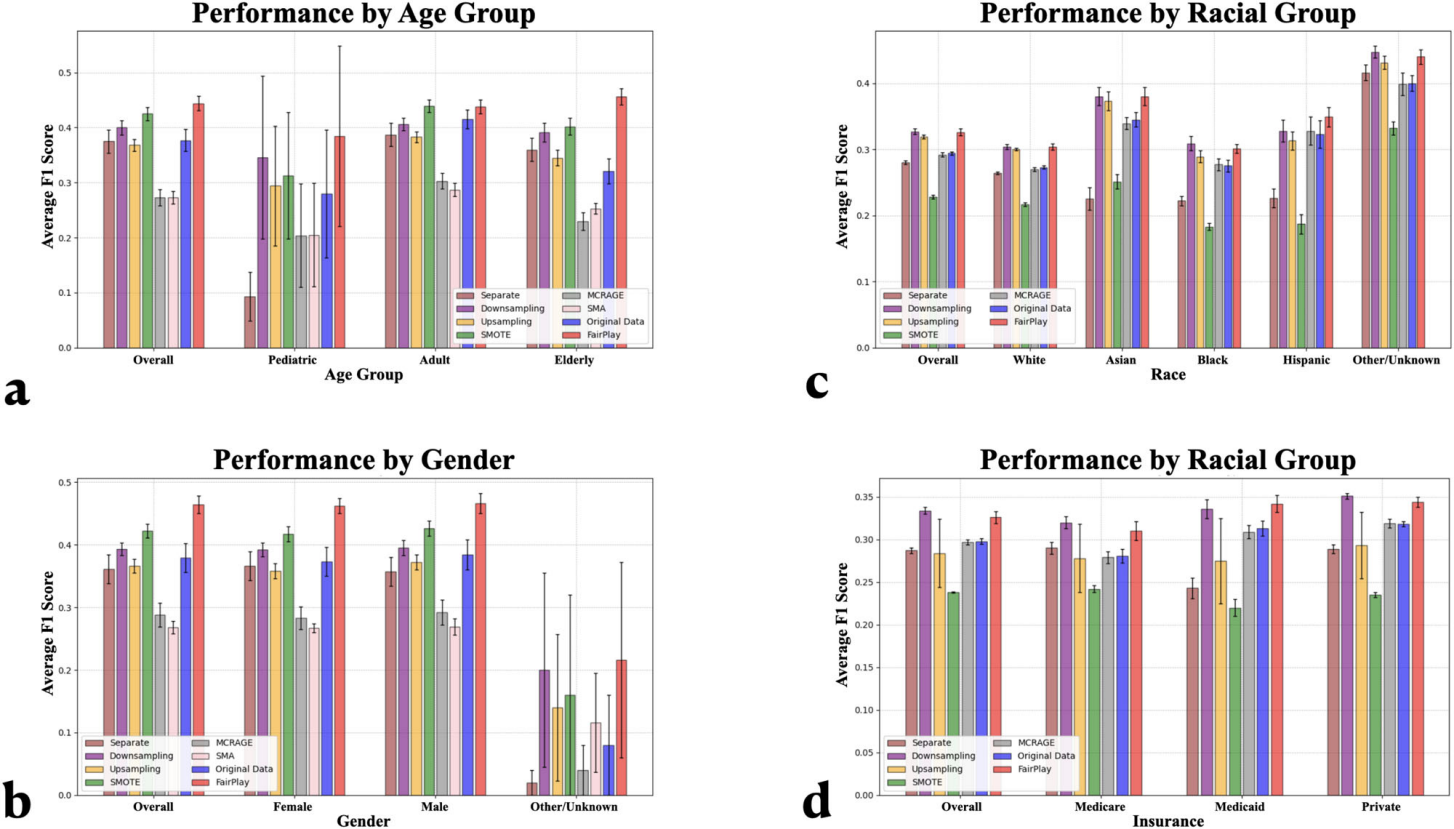
Group-Stratified Test Set Performance: Average F1 score across all downstream models for our compared methods overall and across different subgroups. FairPlay improves performance for each group in each experiment, reducing the disparity in group performances. The figures show performance stratified by **a** age, **b** gender, **c** racial group, and **d** insurance type.

We then showcase the average F1 Score across the downstream models trained on the original data and the data generated by each compared method for each group in each experiment in Fig. 3. A comprehensive set of results for each downstream model is presented individually in Supplementary Tables 2 and 3.

The results indicate an improvement in overall performance within each experiment, similar in magnitude to the previous section, showcasing that FairPlay does not lose its overall performance improvement when it generates conditioned on protected characteristics. However, beyond an improvement in each group population, the disparity in performance among groups is also reduced, especially within the eICU experiments. For example, performance across each age group improves, and crucially FairPlay’s balancing erodes the difference in adult over pediatric and elderly model performance stemming from the overrepresentation of adult and middle-aged patients in the eICU dataset. We then see a similar general improvement and balancing of results in gender groups, improving the performance of Female and Other/Unknown patients. In racial groups, black performance is boosted to surpass the previous mean results. In insurance types, the performance improvement reduced the gap between government sponsored insurance programs and private insurance patients. We note that while the error bounds of the Pediatric age group and Other/Unknown gender group are very large due to the tiny size of these groups, the consistency of the ordering and gaps among compared methods within folds (even as the overall metrics themselves move from fold to fold) along with the improvement of the more prevalent minority groups support this takeaway of general balancing among majority and minority groups.

To make this balancing of results more explicit, we also calculate a pair of fairness metrics for each experiment to showcase that the performance is more equitable. We provide these values in Table 3. There we see that FairPlay is the only compared method to improve both fairness metrics by reducing Theil Index and moving Disparate Impact closer to 1 in all four experiments, and generally improves among the most across methods that do not impede overall performance. These results show that downsampling is generally an effective fairness method, although often at the expense of performance. This trend can be attributed to the fact that in this method, patients are represented equally, but downstream models have less opportunity for capturing trends in training data due to having fewer samples to train on. Additionally, we note that the original disparate impact in the race experiment is positive, owing to the white performance being below the original average. However, that does not limit the value of improving both fairness metrics nor the importance of monitoring poorly performing groups. We provide additional metrics for all four experiments in our supplement as well, where we find similar results of consistent improvement even as we drill down into more specific conditions and aspects of performance.

**Table 3:** Group Fairness Metrics

	eICU				MIMIC-IV			
Training Data	Age		Gender		Race		Insurance	
	Disparate Impact	Theil Index	Disparate Impact	Theil Index	Disparate Impact	Theil Index	Disparate Impact	Theil Index
Original Data	0.772	0.0137	0.971	0.1566	1.217	0.0106	0.907	0.0015
Upsampling	0.898	0.0058	0.962	0.0760	1.165	0.0119	0.946	**0.0004**
Downsampling	0.961	**0.0023**	**0.992**	**0.0419**	1.203	0.0112	0.922	0.0007
Separate Models	0.917	0.1348	1.024	0.3003	**1.083**	0.0342	0.966	0.0033
SMOTE	0.912	0.0097	0.978	0.1233	1.108	0.0260	**1.008**	0.0008
MCRAGE	0.757	0.0142	0.968	0.2100	1.232	0.0104	0.896	0.0016
SMA	0.878	0.0093	**0.992**	0.0606	–	–	–	–
FairPlay	**1.038**	0.0026	0.991	0.0519	1.197	**0.0105**	0.923	0.0011

Bold fonts indicates the best performing model.

Overall, between our performance and fairness metrics, we see that FairPlay provides an improvement across all facets of the study. FairPlay improves overall predictive modeling performance, modeling performance for each subgroup, and facilitates shrinking or even eliminating the disparity in subgroup performance.

### Multi-group performance

**Fig. 4:**
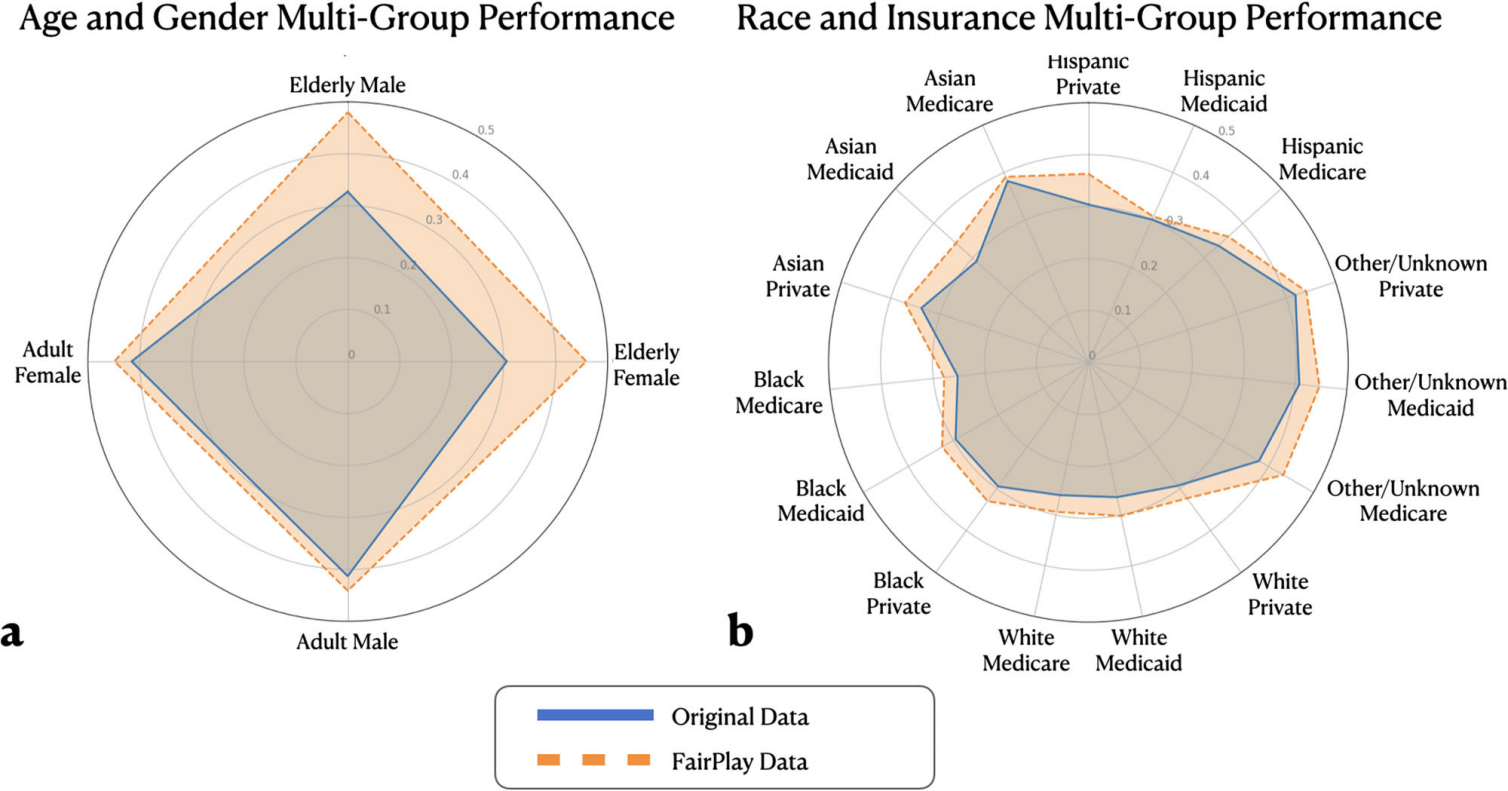
Test Set Stratification Across Multiple Co-Variate Groups: Going beyond subgroups for individual characteristics, we show average F1 score for groups defined by characteristic pairs for both the original and FairPlay-enhanced data. We see both that FairPlay improves performance for each group and generally balances performance across groups, especially on the eICU dataset. The subfigures show performance stratification for (**a**) the eICU inpatient dataset across gender and age groups and (**b**) the MIMIC (outpatient) dataset across insurance types and racial groups.

Finally, we perform a comprehensive, multi-group evaluation for both datasets to demonstrate the capability of FairPlay to learn and debias a large number of protected characteristics at once with complex, multivariate patterns all while maintaining its general performance boost capabilities. To do this, we perform our FairPlay process with all attributes explored as protected characteristics. Specifically, we experimented with the combination of gender and age labels on the eICU dataset and, similarly, with both race and insurance type conditional generation at once on the MIMIC dataset. We present radar plots of performance across all of these group pairs for both the original and FairPlay-augmented training data in Fig. 4. For this experiment, we omitted extremely rare groups with less than 1% representation like pediatric age and unknown gender as they become incredibly small or nonexistent when slicing them further, preventing granular and reliable test set evaluation.

In this experiment, we can see the similar patterns from our first two experimental sections, replicated in this more complex setting. Not only is the FairPlay-augmented performance stronger in general, that performance gain is found in every single subgroup. The performance is also more homogeneous, especially in the eICU age and gender experiment, indicating reduced performance variation among groups. While the original data has substantial discrepancies in performance across different group pairs, FairPlay can balance and debias the data so that the vast majority of populations test within a narrow range of performance. This final experiment is designed to replicate a true production deployment of FairPlay in which a hospital system or other medical entity is concerned with any number of different attributes, and we show that FairPlay is not only capable of handling such complex settings but excels in them.

## Discussion

ML in healthcare faces several innate challenges. Rare positive outcome labels for many tasks are inherently more sensitive and important but more difficult to detect. Populations are often incredibly diverse with respect to both conditions and demographics. Biases in models often stem from factors like the under-representation of certain demographics, disparities in healthcare access, and implicit biases in data collection and analysis. Our work targets these issues by utilizing the HALO^44^ framework to systematically identify and address such challenges by generating synthetic patient data via a powerful, large language model-based approach. This creates a balanced, representative dataset that integrates the unique patterns of diverse groups and outcomes, promoting equitable decision-making and potentially improving outcomes for underrepresented groups. We simulate real-world deployments on a pair of datasets and a suite of experimental setups to show that our FairPlay approach not only diminishes disparities between groups but also boosts overall model performance by equalizing outcome prevalence.

FairPlay can do this in a versatile and scalable manner, leveraging a powerful, large language model-based architecture and performing all pipeline intervention during the preprocessing stage to offer a flexible solution for a variety of dataset shapes, sizes, and types. We show that FairPlay offers impressive consistency, improving performance for each downstream model and increasing fairness across every protected characteristic. However, we can also analyze the types of datasets that are more readily accepting of such improvements. We find that larger disparities in representation yield larger gaps in performance, but groups that are too small incur large amounts of variability. We also find that groups that have more clear and overt differences in their patient data are more likely to yield large performance gaps, which then are both more in need of and also more responsive to this generative balancing. For example, the disparities and fairness improvements in the age and gender experiments were starker than those in the ethnicity and insurance-type experiments. These findings then raise additional questions regarding the possible limitations of applying FairPlay to tiny datasets, rare disease tasks, data with minimal demographic diversity, or purely superficial protected characteristics. Answering these questions is left to further experiments.

We also note that we focus specifically on the ability of models to accurately detect positive labels (e.g., high-risk patients) while simultaneously limiting false positives, as measured by metrics such as F1 Score, when we discuss model performance. Previous work^28,45,46^ has shown that generative models can not yield simultaneous gains in base-level performance (as measured by metrics such as AUROC) and fairness without added power in terms of additional data or weaker downstream models. We find similar results in such AUROC metrics, merely maintaining rather than boosting performance. However, findings in terms of improving fairness and outpacing the performance of other generative models and compared methods are mirrored.

However, a key focus of our experiments is to simulate a real-world hospital setting with disparate tasks, datasets, and downstream classification models. Accordingly, a significant benefit of our FairPlay method is its ability to correct biases in naturally occurring clinical datasets, negating the need for additional training interventions or adjustments to the modeling pipeline. Preventing the need to tune a prediction threshold or utilize more complex models while still optimizing the predictive capabilities and the ability to identify at-risk patients is a large part of that. We show that FairPlay can not only improve the ability of downstream models to do that but also simultaneously normalize the quality of such predictions across groups, downstream models, and predictive pipelines in general. We furthermore note that FairPlay conceptually possesses the capability to expand even further to support multiple tasks and even more groups at once, creating a global model. This can help counteract a limitation where FairPlay is only able to directly debias characteristics which are within the data distribution. While its general representative sampling would not be expected to exacerbate the bias of other protected characteristics, FairPlay can use its scalability to leverage large, multi-dimensional datasets to learn all types of patterns before debiasing for multiple different use cases. Therefore, further exploration and application of this methodology are essential for advancing equitable healthcare.

## Methods

### Background and related work

#### Unfairness in medical machine learning models

Several studies have been conducted on bias produced by medical ML models as a result of unbalanced training datasets. For example^47^, evaluates a system designed to quantify a patient’s degree of clinical health risk based on historical insurance claims. In this study, the authors identify that utilizing health care costs as a proxy for ground truth health outcomes inadequately captures patient treatment needs and ultimately results in unequal access to care for Black patients. Consequently, the authors find that utilizing a label that additionally includes health outcomes resulted in an 84% reduction in bias in the Black patient population. In the task of diagnosing diabetic retinopathy (DR)^48^, formulated the problem of reducing disparate diagnostic accuracy among racial sub-populations as a domain adaptation task. The authors utilize generative methods to construct a synthetic dataset that can be used to train a population-specific diagnostic model for a target population. Similarly^49^, reduces the bias in neural networks trained for image-based DR classification by using adversarial training to produce a neural network whose patient representation suppresses protected characteristics such as race. The de-biased representation is then used to construct an augmented training set.

#### Metrics measuring unfairness

An important first step in solving that unfairness in medical ML models is evaluating and quantifying it. To that end, there has been a lot of work proposing, comparing, and assessing the efficacy of different fairness metrics^50–53^. Many different metrics can be used for many different use cases, corresponding to the fact that different types of disparities are more problematic in different settings. For example, in many medical tasks, different groups need to have the same likelihood of positive classification, which may correspond to receiving additional treatment. In other settings, preventing false positives or having equal overall accuracy may be more critical.

#### Existing approaches

There are then several existing approaches aimed at reducing the performance gap between different groups and improving those metrics. These range from naive approaches such as upsampling data points from minority groups^24–26^, downsampling other data points so that different groups are equally represented^25,29,30^, and training separate models^31^ to more complex ML methods.

The more complex methods then comprise an entire subdomain of ML. These techniques (see surveys^54–57^) fall into the three categories. The first is pre-processing, which removes biases from the training data^58–60^. The second is in-processing, which modifies the model learning process to remove discrimination and encourage fairness^32–37^. Finally, post-processing aims to adjust model outputs to make them fair^61,62^.

### Our proposed FairPlay method

We introduce FairPlay, a significant language model-based pre-processing technique designed to augment and debias datasets. This approach not only bolsters overall performance in label imbalances but also diminishes the propensity for downstream ML models to harbor unfair or biased patterns.

#### Learning dataset patterns

We employ the current state-of-the-art health record generation model, HALO^44^, to learn a mapping $P({\bf{R}}| l,{\mathcal{C}})$ from the outcome label and group memberships to the actual patient record data. HALO is a conditional generative model designed as a significant language model architecture to capture the relationships and patterns within the data over time using stacks of transformer decoder blocks. By representing *l* and ${\mathcal{C}}$ as our combined conditioning information, **i**, and positioning it before the record representation **R** before providing them both as input to the model, we ensure that HALO captures the complex relationships between medical data, protected characteristics, and outcome labels. By learning these patterns, we gain insights into the conditional distributions of the data, learning the divergent patterns between different outcomes and groups so that we may better pinpoint and counteract biases.

#### Analyzing dataset imbalances

To identify and address imbalances in the dataset, both in terms of overall label skew and specific group memberships, we analyze the distribution of data stratified by label and protected characteristics. Specifically, we calculate the distribution and count for each group-label pair within the dataset.

Furthermore, we benchmark existing performance and quantify the unfairness of these imbalances by training a baseline ML model on this initial training dataset, and deploying it on our test set. We compare the performance of different groups in the test set and save these results for future comparison.

#### Generating balanced, augmented datasets

We then use the trained HALO model to sample from the learned probability distribution, $P({\bf{R}}| {\mathcal{C}},{\bf{l}})$, to generate a balanced dataset in terms of both labels and group memberships. Specifically, we identify the most prevalent label-group pair in our training dataset, which has *m* patients. We note that the group here refers to a single assignment of protected characteristics but may correspond to any number of variables. For example, a group could be older women in one dataset or African-American patients in another. For each other label-group pair, with *n* patient records, we create the vector representation of the conditioning information **i** and provide it to the model to simulate *m* − *n* different new patient records. We then combine these synthetic records with the real training data to form our final combined dataset. This process ensures that the resulting dataset is not only balanced with respect to outcome labels to ease the learning process, but it is also balanced with respect to group membership to provide a fair representation of the population and reduce the potential for bias in downstream ML models. Note that while additional tuning can be done regarding the exact degree of balancing (specifically whether *m* − *n* new records are added or some smaller proportion of that), we avoid this tuning for several reasons. First, we aim for FairPlay to be a simple, self-contained process that does not tune, iterate, or interfere with the downstream ML pipeline. Second, this data is designed to be compatible with multiple tasks such that tuning for downstream performance can become complicated. Third, we have found that there is some degree of trade-off between performance and fairness in terms of selecting the optimal amount of additional data to add (and we have included an ablation experiment to this effect shown in Supplementary Fig. 1), so selecting a single point even given the downstream curves may not be a straightforward decision. As such, we have continued here with the straightforward, absolute balancing approach and deferred tuning the amount of synthetic data to further exploration.

#### Training fair models

With the synthetically augmented and balanced dataset in place, we employ the same model training pipeline to retrain the same downstream ML model. By utilizing a more balanced and generally augmented dataset, we aim to achieve improved and fairer results in patient classification without requiring any modifications to the underlying training procedure. This entire process can be seen in Fig. 1.

## Supplementary information

Supplementary information

## Data Availability

MIMIC-IV and eICU datasets can be found at https://physionet.org.

## Code availability

We make our code for all experiments available within the open-source machine learning package for healthcare PyHealth in the pyhealth.synthetic.halo module^63^.
