# Supplementary material for: Improving medical machine learning models with generative balancing for equity and excellence
Source: NPJ Digit Med. 2025 Feb 14;8:100. doi: 10.1038/s41746-025-01438-z (PMC11828851; doi:10.1038/s41746-025-01438-z)
Supplement: Supplementary file 1 — Supplementary information [file 41746_2025_1438_MOESM1_ESM.pdf]

Supplementary Information for "Improving Medical Machine Learning Models with Generative Balancing for Equity and Excellence"

Brandon Theodorou[1], Benjamin Danek[1], Venkat Tummala[1], Shivam Pankaj Kumar[1], Bradley Malin[2] and Jimeng Sun[1*]
University of Illinois at Urbana-Champaign, 201 North Goodwin Avenue, Urbana, IL, USA[1]
Vanderbilt University, 1400 18th Ave S, Nashville, TN, USA[2]
* To whom correspondence should be addressed: jimeng@illinois.edu

We design our main paper to be self-contained and easy to understand both methodologically and experimentally by providing a concise, detailed account of our task, the proposed `MedGenBoost` method, and the experimental results. However, to ensure full completeness and clarity, we have included additional and fully comprehensive experimental details and results in this supplement.

1 SUPPLEMENTARY METHODS

1.1 Group Definitions

Here we define the specific definitions and mappings for our two grouping-based protected characteristics which combine multiple specific values into broader categories. First, for our age experiments, we consider patients pediatric if they are 18 years old or below. We then set the cut off for elderly patients as 75 years or above, and we label all other patients as adult. Second, for our racial groups, we use the mappings from specific entries in the corresponding MIMIC-IV [1] column to broader experimental groups found in Supplementary Table 1.

Supplementary Table 1: Racial Group Mappings

Group	Specific Values
White	White, White - Eastern European, White - Other European, Portuguese, White - Brazilian, White - Russian
Black	Black/African American, Black/Caribbean Island, Black/African, Black/Cape Verdean
Hispanic	Hispanic/Latino - Columbian, South American, Hispanic/Latino - Guatemalan, Hispanic/Latino - Puerto Rican, Hispanic/Latino - Mexican, Hispanic/Latino - Central American, Hispanic/Latino - Honduran, Hispanic/Latino - Cuban, Hispanic/Latino - Dominican, Hispanic or Latino, Hispanic/Latino - Salvadoran
Asian	Asian - South East Asian, Asian - Korean, Asian, Asian - Asian Indian, Asian - Chinese
Other/Unknown	Unable to Obtain, American Indian/Alaska Native, Multiple Race/Ethnicity, Native Hawaiian or Other Pacific Islander, Unknown, Patient Declined to Answer, Other

1.2 Full Hyperparameters

Here provide a comprehensive set of model hyperparameters for full reproducibility in addition to the experimental setup and hyperparameters provided in our main paper. These hyperparameters are largely copied from [2], but we include them here for completeness. The model has 12 coarse layers and 2 fine layers with an internal embedding dimension of 768. We train with a batch size of 512 for the eICU dataset and 256 for the MIMIC dataset (due to memory constraints) for a maximum of 1,000 epochs and a patience parameter of 3, based on an evaluation performed on the validation set once an epoch. Note that we never come close to using the full 1,000 epochs, generally concluding in less than 100 epochs.

2 SUPPLEMENTARY RESULTS

2.1 Full Group Experiment Results

While we provided group-by-group results for each compared method averaged over our downstream models in our main paper, we provide the F1 Scores for each downstream model individually in Supplementary Table 2 for the two eICU dataset experiments and Supplementary Table 3 for the two MIMIC-IV dataset experiments.

2.2 Additional Fairness Metrics

For the purposes of conciseness and clarity, we focus on two fairness metrics, Disparate Impact, and Theil Index, in our main paper, which provide broad measures of fairness. However, fairness is a multifaceted and well-studied concept with many additional metrics measuring more specific sub-metrics such as false positive rate. So, we include four additional metrics here. First, we present *Performance Variance*, which is similar to Theil Index but uses a more simple variance calculation over the score distribution. For that metric, lower scores are better. Next, we use *Equality of Opportunity*, which is the ratio of true positive rate (recall) in minority groups compared to in the majority group. *Equalized Odds* is then a step further, looking at both true positive rate and false positive rate and providing the lower of the two ratios. Finally, *Demographic Parity* looks at the ratio of positive predictions more generally. Each of these latter three metrics is aimed at ensuring a similar distribution of predictions (and thus the allocation of resources) across groups, and given their presentation as ratios (as opposed to differences), scores closest to 1 are best. We present these results in Table 4 where we see similar results as in our main paper (albeit with slightly less consistency as these metrics can be more sensitive to differences in label prevalence across groups). More of our compared methods improve upon baseline data in the majority of metrics across each

Supplementary Table 2: eICU Group Prediction Performance

Experiment	Subgroup	Training Data	Downstream Method F1 Score					
			Avg	KNN	LR	NN	RFC	XGB
Age	Overall	Original Data	0.377 ± 0.02	0.177 ± 0.01	0.453 ± 0.03	**0.495 ± 0.02**	0.268 ± 0.02	0.492 ± 0.02
		Upsampling	0.368 ± 0.01	0.228 ± 0.02	**0.473 ± 0.01**	0.441 ± 0.02	0.194 ± 0.02	0.504 ± 0.01
		Downsampling	0.4 ± 0.01	0.198 ± 0.03	0.419 ± 0.01	0.457 ± 0.01	**0.47 ± 0.01**	0.455 ± 0.01
		Separate	0.375 ± 0.02	0.168 ± 0.02	0.46 ± 0.03	0.492 ± 0.03	0.263 ± 0.02	0.492 ± 0.02
		SMOTE	0.425 ± 0.01	0.275 ± 0.02	0.471 ± 0.01	0.454 ± 0.02	0.419 ± 0.02	**0.505 ± 0.01**
		MCRAGE	0.273 ± 0.01	0.129 ± 0.01	0.011 ± 0.01	0.488 ± 0.02	0.296 ± 0.02	0.442 ± 0.02
		SMA	0.273 ± 0.01	0.082 ± 0.01	0.403 ± 0.01	0.413 ± 0.02	0.091 ± 0.01	0.376 ± 0.01
		MedGenBoost	**0.444 ± 0.01**	**0.323 ± 0.02**	0.472 ± 0.01	0.486 ± 0.01	0.437 ± 0.01	0.502 ± 0.01
	Pediatric	Original Data	0.28 ± 0.12	0.133 ± 0.13	**0.42 ± 0.18**	0.422 ± 0.19	0.057 ± 0.06	0.367 ± 0.15
		Upsampling	0.294 ± 0.11	0.1 ± 0.1	0.412 ± 0.2	0.36 ± 0.22	0.0 ± 0.0	**0.598 ± 0.18**
		Downsampling	0.346 ± 0.15	0.257 ± 0.11	0.385 ± 0.18	**0.427 ± 0.2**	0.3 ± 0.12	0.363 ± 0.16
		Separate	0.093 ± 0.04	0.0 ± 0.0	0.233 ± 0.14	0.0 ± 0.0	0.0 ± 0.0	0.233 ± 0.14
		SMOTE	0.313 ± 0.12	0.137 ± 0.09	0.385 ± 0.19	0.233 ± 0.14	0.257 ± 0.11	0.551 ± 0.18
		MCRAGE	0.204 ± 0.09	0.133 ± 0.13	0.0 ± 0.0	0.362 ± 0.17	0.19 ± 0.13	**0.333 ± 0.14**
		SMA	0.205 ± 0.09	0.08 ± 0.08	0.343 ± 0.17	0.312 ± 0.14	0.057 ± 0.06	0.233 ± 0.14
		MedGenBoost	**0.384 ± 0.16**	**0.333 ± 0.14**	**0.42 ± 0.19**	0.381 ± 0.2	**0.325 ± 0.16**	0.462 ± 0.17
	Adult	Original Data	0.415 ± 0.02	0.206 ± 0.02	**0.5 ± 0.03**	**0.535 ± 0.02**	0.307 ± 0.02	**0.531 ± 0.02**
		Upsampling	0.383 ± 0.01	0.258 ± 0.02	0.473 ± 0.01	0.464 ± 0.01	0.218 ± 0.02	0.505 ± 0.0
		Downsampling	0.406 ± 0.01	0.239 ± 0.03	0.415 ± 0.01	0.449 ± 0.01	**0.477 ± 0.01**	0.449 ± 0.0
		Separate	0.387 ± 0.02	0.184 ± 0.02	0.474 ± 0.03	0.51 ± 0.03	0.249 ± 0.02	0.518 ± 0.01
		SMOTE	**0.439 ± 0.01**	0.299 ± 0.02	0.461 ± 0.01	0.478 ± 0.02	0.459 ± 0.02	0.499 ± 0.01
		MCRAGE	0.303 ± 0.01	0.156 ± 0.02	0.015 ± 0.01	0.522 ± 0.02	0.338 ± 0.02	0.485 ± 0.02
		SMA	0.287 ± 0.01	0.089 ± 0.01	0.408 ± 0.02	0.433 ± 0.02	0.107 ± 0.01	0.397 ± 0.01
		MedGenBoost	0.438 ± 0.01	**0.349 ± 0.02**	0.46 ± 0.01	0.472 ± 0.02	0.415 ± 0.02	0.495 ± 0.01
	Elderly	Original Data	0.321 ± 0.02	0.136 ± 0.01	0.382 ± 0.03	0.435 ± 0.03	0.215 ± 0.02	0.435 ± 0.03
		Upsampling	0.345 ± 0.01	0.182 ± 0.02	0.472 ± 0.01	0.405 ± 0.02	0.162 ± 0.02	0.502 ± 0.01
		Downsampling	0.391 ± 0.02	0.135 ± 0.03	0.423 ± 0.02	0.47 ± 0.01	0.459 ± 0.02	0.465 ± 0.01
		Separate	0.36 ± 0.02	0.149 ± 0.01	0.441 ± 0.03	0.471 ± 0.03	0.281 ± 0.02	0.459 ± 0.02
		SMOTE	0.402 ± 0.01	0.237 ± 0.02	0.487 ± 0.01	0.417 ± 0.02	0.356 ± 0.03	**0.515 ± 0.01**
		SMA	0.253 ± 0.01	0.072 ± 0.01	0.395 ± 0.01	0.384 ± 0.02	0.07 ± 0.01	0.346 ± 0.02
		MCRAGE	0.23 ± 0.02	0.091 ± 0.01	0.006 ± 0.0	0.437 ± 0.02	0.237 ± 0.03	0.378 ± 0.03
		MedGenBoost	**0.456 ± 0.01**	**0.283 ± 0.03**	**0.492 ± 0.01**	**0.511 ± 0.01**	**0.477 ± 0.01**	**0.515 ± 0.01**
Gender	Overall	Original Data	0.379 ± 0.02	0.176 ± 0.02	0.452 ± 0.03	0.501 ± 0.03	0.269 ± 0.02	0.496 ± 0.02
		Upsampling	0.366 ± 0.01	0.247 ± 0.02	0.452 ± 0.01	0.43 ± 0.01	0.21 ± 0.02	0.492 ± 0.01
		Downsampling	0.393 ± 0.01	0.21 ± 0.03	0.406 ± 0.01	0.432 ± 0.02	0.469 ± 0.01	0.45 ± 0.01
		Separate	0.361 ± 0.02	0.153 ± 0.02	0.453 ± 0.03	0.483 ± 0.03	0.24 ± 0.02	0.477 ± 0.02
		SMOTE	0.422 ± 0.01	0.287 ± 0.02	0.459 ± 0.01	0.445 ± 0.01	0.423 ± 0.02	0.497 ± 0.0
		SMA	0.268 ± 0.01	0.094 ± 0.01	0.376 ± 0.01	0.429 ± 0.02	0.082 ± 0.01	0.36 ± 0.01
		MCRAGE	0.288 ± 0.02	0.129 ± 0.01	0.071 ± 0.02	0.489 ± 0.02	0.296 ± 0.02	0.452 ± 0.02
		MedGenBoost	**0.464 ± 0.01**	**0.311 ± 0.02**	**0.504 ± 0.01**	**0.503 ± 0.02**	**0.481 ± 0.01**	**0.521 ± 0.01**
	Female	Original Data	0.373 ± 0.02	0.169 ± 0.02	0.45 ± 0.03	0.498 ± 0.03	0.259 ± 0.02	0.489 ± 0.02
		Upsampling	0.358 ± 0.01	0.243 ± 0.02	0.445 ± 0.01	0.421 ± 0.02	0.2 ± 0.02	0.483 ± 0.01
		Downsampling	0.392 ± 0.01	0.206 ± 0.03	0.399 ± 0.01	0.434 ± 0.02	0.47 ± 0.01	0.448 ± 0.01
		Separate	0.366 ± 0.02	0.16 ± 0.03	0.459 ± 0.03	0.492 ± 0.02	0.239 ± 0.03	0.479 ± 0.02
		SMOTE	0.417 ± 0.01	0.274 ± 0.01	0.455 ± 0.01	0.439 ± 0.02	0.421 ± 0.03	0.496 ± 0.01
		SMA	0.267 ± 0.01	0.091 ± 0.01	0.38 ± 0.01	0.433 ± 0.02	0.076 ± 0.01	0.355 ± 0.01
		MCRAGE	0.283 ± 0.02	0.123 ± 0.02	0.066 ± 0.02	0.484 ± 0.02	0.289 ± 0.03	0.452 ± 0.02
		MedGenBoost	**0.462 ± 0.01**	**0.3 ± 0.02**	**0.503 ± 0.01**	**0.505 ± 0.01**	**0.48 ± 0.01**	**0.521 ± 0.01**
	Male	Original Data	0.384 ± 0.02	0.182 ± 0.02	0.453 ± 0.03	**0.503 ± 0.03**	0.278 ± 0.02	0.502 ± 0.03
		Upsampling	0.372 ± 0.01	0.251 ± 0.03	0.457 ± 0.01	0.437 ± 0.02	0.218 ± 0.01	0.499 ± 0.01
		Downsampling	0.395 ± 0.01	0.214 ± 0.04	0.412 ± 0.01	0.43 ± 0.02	0.467 ± 0.01	0.452 ± 0.01
		Separate	0.357 ± 0.02	0.147 ± 0.01	0.448 ± 0.03	0.476 ± 0.04	0.241 ± 0.02	0.474 ± 0.02
		SMOTE	0.426 ± 0.01	0.297 ± 0.03	0.463 ± 0.01	0.45 ± 0.01	0.424 ± 0.02	0.499 ± 0.01
		SMA	0.269 ± 0.01	0.097 ± 0.01	0.372 ± 0.01	0.426 ± 0.03	0.086 ± 0.01	0.364 ± 0.02
		MCRAGE	0.292 ± 0.02	0.135 ± 0.01	0.075 ± 0.03	0.494 ± 0.02	0.303 ± 0.02	0.453 ± 0.03
		MedGenBoost	**0.466 ± 0.02**	**0.32 ± 0.02**	**0.506 ± 0.01**	0.501 ± 0.02	**0.482 ± 0.02**	**0.521 ± 0.02**
	Other/Unknown	Original Data	0.08 ± 0.08	0.0 ± 0.0	0.0 ± 0.0	0.2 ± 0.2	0.0 ± 0.0	**0.2 ± 0.2**
		Upsampling	0.14 ± 0.12	0.0 ± 0.0	**0.3 ± 0.2**	0.2 ± 0.2	0.0 ± 0.0	**0.2 ± 0.2**
		Downsampling	0.2 ± 0.15	0.0 ± 0.0	**0.3 ± 0.2**	**0.3 ± 0.2**	0.2 ± 0.2	**0.2 ± 0.2**
		Separate	0.02 ± 0.02	0.0 ± 0.0	0.0 ± 0.0	0.0 ± 0.0	0.0 ± 0.0	0.1 ± 0.1
		SMOTE	0.16 ± 0.16	0.0 ± 0.0	0.2 ± 0.2	0.2 ± 0.2	0.2 ± 0.2	**0.2 ± 0.2**
		SMA	0.116 ± 0.08	0.0 ± 0.0	**0.314 ± 0.2**	0.2 ± 0.2	0.0 ± 0.0	0.067 ± 0.07
		MCRAGE	0.04 ± 0.04	0.0 ± 0.0	0.0 ± 0.0	0.2 ± 0.2	0.0 ± 0.0	0.0 ± 0.0
		MedGenBoost	**0.216 ± 0.16**	0.0 ± 0.0	0.3 ± 0.2	**0.3 ± 0.2**	**0.28 ± 0.2**	**0.2 ± 0.2**

Supplementary Table 3: MIMIC Group Prediction Performance

Experiment	Subgroup	Training Data	Downstream Method F1 Score					
			Avg	KNN	LR	NN	RFC	XGB
Race	Overall	Original Data	0.294 ± 0.0	0.112 ± 0.0	0.388 ± 0.0	0.417 ± 0.01	0.153 ± 0.0	0.401 ± 0.0
		Upsampling	0.319 ± 0.0	**0.198 ± 0.0**	**0.414 ± 0.0**	0.393 ± 0.0	0.161 ± 0.0	**0.427 ± 0.0**
		Downsampling	**0.327 ± 0.0**	0.136 ± 0.0	**0.414 ± 0.0**	0.421 ± 0.01	0.251 ± 0.01	0.415 ± 0.0
		Separate	0.28 ± 0.0	0.099 ± 0.0	0.39 ± 0.01	0.393 ± 0.0	0.128 ± 0.0	0.391 ± 0.0
		SMOTE	0.228 ± 0.0	0.143 ± 0.01	0.343 ± 0.0	0.212 ± 0.01	0.047 ± 0.0	0.397 ± 0.0
		MCRAGE	0.292 ± 0.0	0.103 ± 0.0	0.356 ± 0.01	0.427 ± 0.01	0.178 ± 0.0	0.397 ± 0.01
		MedGenBoost	0.326 ± 0.01	0.131 ± 0.0	0.396 ± 0.0	**0.436 ± 0.01**	**0.267 ± 0.01**	0.399 ± 0.01
	White	Original Data	0.273 ± 0.0	0.096 ± 0.0	0.37 ± 0.0	0.39 ± 0.01	0.133 ± 0.0	0.377 ± 0.0
		Upsampling	0.3 ± 0.0	**0.187 ± 0.0**	**0.396 ± 0.0**	0.375 ± 0.0	0.137 ± 0.0	**0.403 ± 0.0**
		Downsampling	**0.304 ± 0.0**	0.12 ± 0.0	0.394 ± 0.0	0.395 ± 0.01	0.219 ± 0.0	0.393 ± 0.01
		Separate	0.264 ± 0.0	0.09 ± 0.0	0.371 ± 0.01	0.377 ± 0.0	0.11 ± 0.0	0.372 ± 0.0
		SMOTE	0.217 ± 0.0	0.128 ± 0.01	0.34 ± 0.0	0.187 ± 0.01	0.039 ± 0.0	0.39 ± 0.01
		MCRAGE	0.27 ± 0.0	0.088 ± 0.0	0.336 ± 0.01	0.402 ± 0.01	0.151 ± 0.0	0.373 ± 0.01
		MedGenBoost	**0.304 ± 0.01**	0.116 ± 0.0	0.376 ± 0.0	**0.408 ± 0.01**	**0.241 ± 0.01**	0.376 ± 0.01
	Black	Original Data	0.275 ± 0.01	0.084 ± 0.01	0.369 ± 0.02	0.399 ± 0.02	0.132 ± 0.01	0.39 ± 0.01
		Upsampling	0.289 ± 0.01	**0.148 ± 0.01**	0.38 ± 0.01	0.358 ± 0.02	0.157 ± 0.01	**0.401 ± 0.01**
		Downsampling	**0.309 ± 0.01**	0.127 ± 0.02	**0.397 ± 0.01**	0.398 ± 0.02	0.234 ± 0.01	0.392 ± 0.02
		Separate	0.222 ± 0.01	0.052 ± 0.01	0.349 ± 0.01	0.334 ± 0.02	0.035 ± 0.01	0.342 ± 0.01
		SMOTE	0.183 ± 0.01	0.112 ± 0.01	0.269 ± 0.01	0.179 ± 0.01	0.025 ± 0.0	0.33 ± 0.01
		MCRAGE	0.277 ± 0.01	0.079 ± 0.01	0.347 ± 0.02	0.409 ± 0.01	0.170 ± 0.01	0.377 ± 0.01
		MedGenBoost	0.301 ± 0.01	0.112 ± 0.01	0.366 ± 0.02	**0.416 ± 0.01**	**0.247 ± 0.01**	0.366 ± 0.01
	Hispanic	Original Data	0.323 ± 0.02	0.119 ± 0.01	0.419 ± 0.02	0.46 ± 0.04	0.17 ± 0.02	0.449 ± 0.03
		Upsampling	0.313 ± 0.01	**0.162 ± 0.02**	**0.421 ± 0.02**	0.374 ± 0.01	0.164 ± 0.02	0.443 ± 0.03
		Downsampling	0.328 ± 0.02	0.122 ± 0.01	0.384 ± 0.02	0.438 ± 0.01	0.257 ± 0.03	0.437 ± 0.03
		Separate	0.226 ± 0.01	0.063 ± 0.01	0.379 ± 0.02	0.314 ± 0.03	0.021 ± 0.01	0.355 ± 0.03
		SMOTE	0.187 ± 0.01	0.117 ± 0.01	0.252 ± 0.02	0.196 ± 0.05	0.045 ± 0.01	0.327 ± 0.02
		MCRAGE	0.328 ± 0.02	0.124 ± 0.01	0.402 ± 0.02	0.459 ± 0.03	0.192 ± 0.03	**0.461 ± 0.03**
		MedGenBoost	**0.349 ± 0.01**	0.157 ± 0.01	0.419 ± 0.02	**0.465 ± 0.02**	**0.291 ± 0.02**	0.414 ± 0.02
	Asian	Original Data	0.345 ± 0.01	0.118 ± 0.02	0.44 ± 0.01	0.49 ± 0.01	0.208 ± 0.02	0.466 ± 0.01
		Upsampling	0.373 ± 0.01	**0.249 ± 0.02**	0.468 ± 0.01	0.442 ± 0.02	0.221 ± 0.02	**0.485 ± 0.03**
		Downsampling	**0.38 ± 0.01**	0.14 ± 0.01	**0.494 ± 0.01**	0.503 ± 0.02	0.295 ± 0.02	0.466 ± 0.01
		Separate	0.225 ± 0.02	0.046 ± 0.02	0.405 ± 0.01	0.233 ± 0.06	0.077 ± 0.02	0.365 ± 0.01
		SMOTE	0.251 ± 0.01	0.174 ± 0.02	0.368 ± 0.01	0.242 ± 0.03	0.066 ± 0.03	0.403 ± 0.02
		MCRAGE	0.339 ± 0.01	0.11 ± 0.01	0.409 ± 0.02	0.485 ± 0.01	0.233 ± 0.01	0.457 ± 0.01
		MedGenBoost	**0.38 ± 0.01**	0.15 ± 0.02	0.447 ± 0.02	**0.513 ± 0.02**	**0.312 ± 0.01**	0.476 ± 0.03
	Other/Unknown	Original Data	0.4 ± 0.01	0.21 ± 0.01	0.479 ± 0.02	0.539 ± 0.01	0.262 ± 0.01	0.508 ± 0.02
		Upsampling	0.431 ± 0.01	**0.296 ± 0.01**	0.517 ± 0.01	0.515 ± 0.01	0.278 ± 0.02	**0.551 ± 0.01**
		Downsampling	**0.447 ± 0.01**	0.228 ± 0.01	**0.521 ± 0.01**	0.548 ± 0.01	**0.407 ± 0.02**	0.531 ± 0.01
		Separate	0.416 ± 0.01	0.2 ± 0.01	0.508 ± 0.02	0.545 ± 0.01	0.305 ± 0.02	0.524 ± 0.01
		SMOTE	0.332 ± 0.01	0.239 ± 0.02	0.45 ± 0.01	0.361 ± 0.02	0.104 ± 0.01	0.506 ± 0.01
		MCRAGE	0.399 ± 0.02	0.193 ± 0.01	0.44 ± 0.02	0.55 ± 0.02	0.304 ± 0.02	0.509 ± 0.02
		MedGenBoost	0.44 ± 0.01	0.215 ± 0.01	0.5 ± 0.01	**0.564 ± 0.01**	0.401 ± 0.02	0.519 ± 0.01
Insurance	Overall	Original Data	0.298 ± 0.0	0.112 ± 0.0	0.388 ± 0.0	0.438 ± 0.01	0.154 ± 0.0	0.4 ± 0.0
		Upsampling	0.284 ± 0.04	**0.181 ± 0.02**	0.371 ± 0.05	0.354 ± 0.04	0.139 ± 0.03	0.376 ± 0.05
		Downsampling	**0.334 ± 0.0**	0.152 ± 0.01	**0.42 ± 0.0**	0.43 ± 0.01	0.251 ± 0.01	**0.417 ± 0.0**
		Separate	0.287 ± 0.0	0.103 ± 0.0	0.391 ± 0.01	0.409 ± 0.01	0.137 ± 0.0	0.396 ± 0.0
		SMOTE	0.238 ± 0.0	0.158 ± 0.01	0.35 ± 0.0	0.208 ± 0.0	0.072 ± 0.0	0.401 ± 0.0
		MCRAGE	0.297 ± 0.0	0.103 ± 0.0	0.385 ± 0.0	0.415 ± 0.01	0.176 ± 0.0	0.407 ± 0.0
		MedGenBoost	0.326 ± 0.01	0.127 ± 0.0	0.398 ± 0.01	**0.441 ± 0.01**	**0.257 ± 0.01**	0.408 ± 0.01
	Private	Original Data	0.318 ± 0.0	0.136 ± 0.01	0.402 ± 0.0	**0.457 ± 0.01**	0.171 ± 0.0	0.424 ± 0.01
		Upsampling	0.293 ± 0.04	**0.188 ± 0.02**	0.379 ± 0.05	0.358 ± 0.04	0.149 ± 0.04	0.393 ± 0.05
		Downsampling	**0.351 ± 0.0**	0.174 ± 0.01	**0.432 ± 0.0**	0.441 ± 0.01	0.271 ± 0.0	0.435 ± 0.0
		Separate	0.289 ± 0.01	0.108 ± 0.01	0.393 ± 0.01	0.423 ± 0.01	0.111 ± 0.0	0.408 ± 0.0
		SMOTE	0.235 ± 0.0	0.164 ± 0.0	0.326 ± 0.0	0.222 ± 0.01	0.083 ± 0.0	0.38 ± 0.0
		MCRAGE	0.319 ± 0.01	0.128 ± 0.01	0.399 ± 0.01	0.437 ± 0.01	0.195 ± 0.0	**0.436 ± 0.01**
		MedGenBoost	0.344 ± 0.01	0.147 ± 0.01	0.411 ± 0.01	0.456 ± 0.01	**0.277 ± 0.01**	0.43 ± 0.01
	Medicare	Original Data	0.281 ± 0.01	0.091 ± 0.01	0.379 ± 0.01	0.422 ± 0.01	0.136 ± 0.01	0.38 ± 0.01
		Upsampling	0.278 ± 0.04	**0.175 ± 0.02**	0.367 ± 0.05	0.353 ± 0.04	0.129 ± 0.03	0.364 ± 0.05
		Downsampling	**0.32 ± 0.01**	0.132 ± 0.01	**0.413 ± 0.01**	0.422 ± 0.01	0.232 ± 0.01	0.403 ± 0.0
		Separate	0.29 ± 0.01	0.1 ± 0.01	0.394 ± 0.01	0.406 ± 0.01	0.16 ± 0.01	0.39 ± 0.01
		SMOTE	0.242 ± 0.0	0.151 ± 0.01	0.379 ± 0.0	0.199 ± 0.0	0.061 ± 0.0	**0.421 ± 0.01**
		MCRAGE	0.279 ± 0.01	0.081 ± 0.01	0.375 ± 0.01	0.397 ± 0.01	0.159 ± 0.01	0.384 ± 0.01
		MedGenBoost	0.31 ± 0.01	0.107 ± 0.01	0.387 ± 0.01	**0.428 ± 0.01**	**0.238 ± 0.01**	0.39 ± 0.01
	Medicaid	Original Data	0.313 ± 0.01	0.131 ± 0.01	0.382 ± 0.02	0.444 ± 0.01	0.193 ± 0.0	**0.414 ± 0.01**
		Upsampling	0.275 ± 0.05	**0.177 ± 0.03**	0.347 ± 0.06	0.327 ± 0.05	0.161 ± 0.04	0.364 ± 0.07
		Downsampling	0.336 ± 0.01	**0.177 ± 0.02**	0.395 ± 0.01	0.419 ± 0.01	0.276 ± 0.02	0.411 ± 0.01
		Separate	0.243 ± 0.01	0.094 ± 0.01	0.35 ± 0.01	0.32 ± 0.03	0.084 ± 0.01	0.365 ± 0.01
		SMOTE	0.22 ± 0.01	0.165 ± 0.01	0.28 ± 0.01	0.193 ± 0.01	0.095 ± 0.01	0.368 ± 0.01
		MCRAGE	0.309 ± 0.01	0.126 ± 0.01	0.377 ± 0.02	0.426 ± 0.01	0.208 ± 0.01	0.41 ± 0.01
		MedGenBoost	**0.342 ± 0.01**	0.157 ± 0.01	**0.406 ± 0.01**	**0.446 ± 0.01**	**0.289 ± 0.01**	**0.414 ± 0.01**

experiment. This includes MedGenBoost which improves in 13 out of 16 metrics across the experiments.

2.3 Degree of Generative Balancing

We made an argument in our main paper for generating the specific number of synthetic patients to perform simple, absolute balancing in terms of different group-outcome pairs. Beyond the conceptual goal of offering flexible, multi-task support and avoiding interference with the downstream machine learning pipeline, we also referenced a performance-fairness trade-off in terms of the amount of synthetic data added. To demonstrate that trade-off, we conducted ablations on our two eICU group experiments of gender and age. Specifically, we added our synthetic data to the original baseline dataset in 10% increments, and we compared both the downstream F1 Score and Disparate Impact at each point. We present these results in Figure 1 where we see that there is indeed such a trade-off. Specifically, we see that downstream performance generally peaks as roughly half of the synthetic data is added before plateauing or even decaying slightly. However, fairness generally continues to improve all the way to the end as more synthetic data is added. This demonstrates both the trade-off itself as well as the challenge of choosing the ideal point along the curves, even before considering that the optimal point is likely to vary by experiment.

SUPPLEMENTARY REFERENCES

[1] Johnson, A. E., Bulgarelli, L., Shen, L., Gayles, A., Shammout, A., Horng, S., Pollard, T. J., Hao, S., Moody, B., Gow, B., et al. Mimic-iv, a freely accessible electronic health record dataset. *Scientific data 10*, 1 (2023), 1.

[2] Theodorou, B., Xiao, C., and Sun, J. Synthesize high-dimensional longitudinal electronic health records via hierarchical autoregressive language model. *Nature Communications 14*, 1 (2023), 5305.

Supplementary Table 4: Additional Fairness Metrics

Experiment	Training Data	Metric<br>Performance Variance	 Equalized Odds	 Equality of Opportunity	 Demographic Parity
Age	Original Data	0.057	0.676	0.683	1.214
	Upsampling	0.036	0.762	0.771	1.286
	Downsampling	**0.025**	0.784	0.793	1.276
	Separate	0.137	**0.900**	**0.900**	1.761
	SMOTE	0.053	0.763	0.771	1.265
	MCRAGE	0.042	0.686	0.688	1.243
	SMA	0.034	0.757	0.757	**1.211**
	`MedGenBoost`	0.031	0.810	0.816	1.243
Gender	Original Data	0.141	0.888	0.957	0.956
	Upsampling	0.106	0.939	0.939	0.951
	Downsampling	0.091	0.953	0.968	0.968
	Separate	0.161	1.026	1.026	1.066
	SMOTE	0.123	0.956	0.956	0.967
	MCRAGE	0.117	0.833	0.955	0.942
	SMA	**0.072**	**0.995**	**0.995**	1.027
	`MedGenBoost`	0.117	0.965	0.973	**0.978**
Race	Original Data	**0.047**	**1.034**	1.288	1.029
	Upsampling	0.053	0.898	1.239	0.968
	Downsampling	0.054	0.963	1.298	1.027
	Separate	0.074	1.108	1.183	1.053
	SMOTE	0.055	0.814	**1.104**	0.866
	MCRAGE	**0.047**	0.900	1.300	**1.020**
	`MedGenBoost`	0.052	0.951	1.287	1.025
Insurance	Original Data	0.016	0.830	0.878	2.055
	Upsampling	**0.008**	0.877	0.894	1.893
	Downsampling	0.017	0.850	0.871	1.890
	Separate	0.022	**0.934**	**0.948**	2.319
	SMOTE	0.009	0.919	0.941	**1.793**
	MCRAGE	0.017	0.821	0.866	2.007
	`MedGenBoost`	0.016	0.856	0.883	2.018

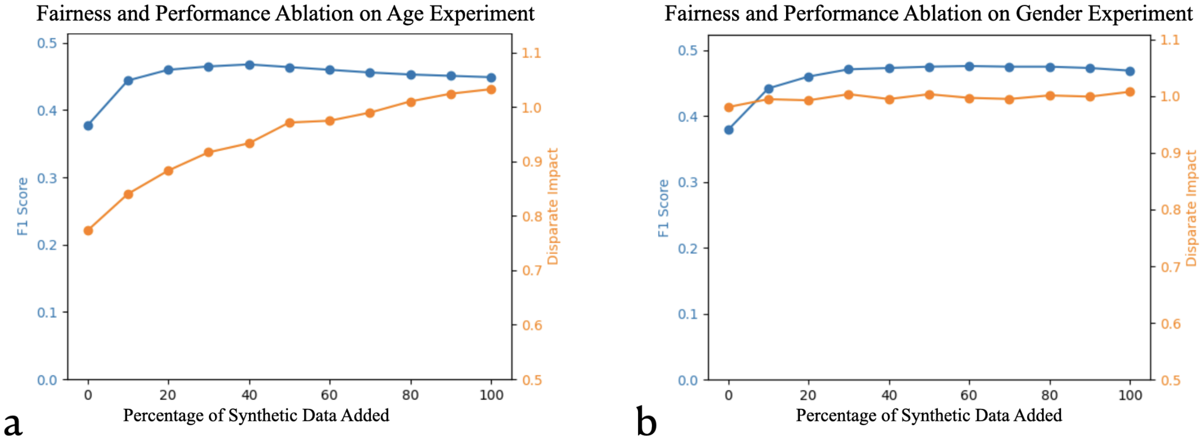

Supplementary Figure 1: The Effect of Synthetic Data Quantity on Performance and Fairness: Ablation results comparing how downstream F1 score and Disparate impact change as we add additional `MedGenBoost` synthetic data in 10% increments. We show this for a) the eICU age experiment, and b) the eICU gender experiment

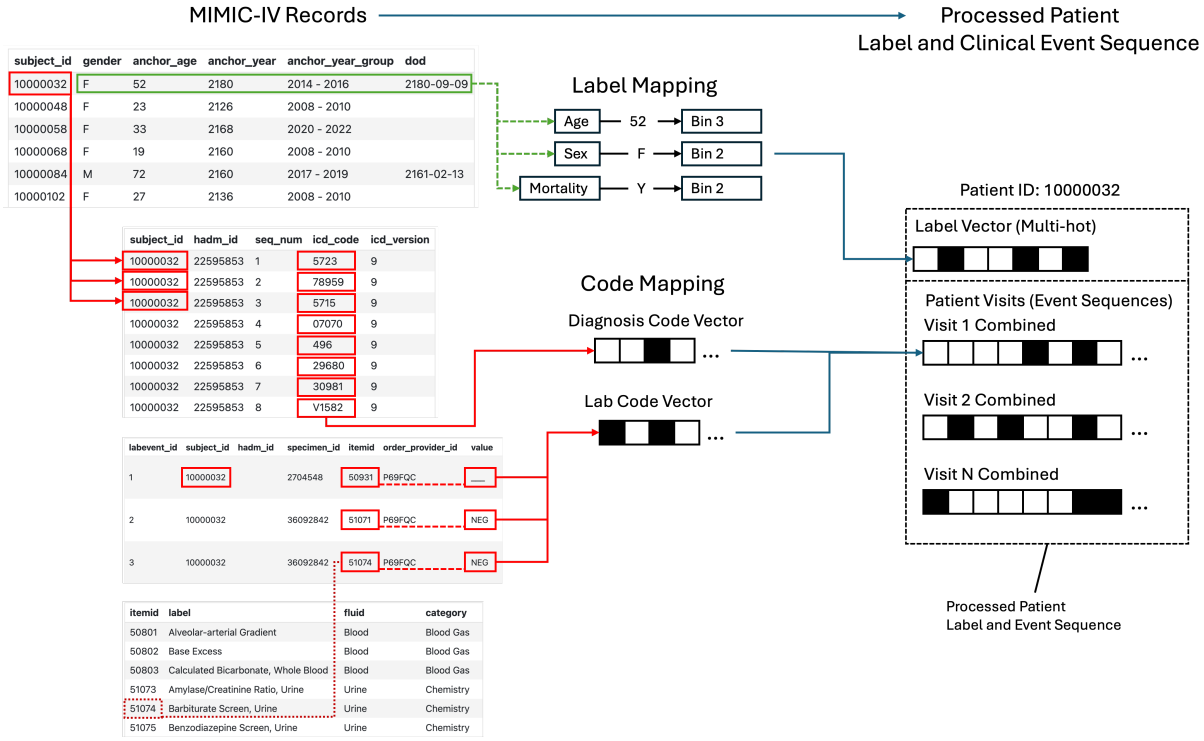

Supplementary Figure 2: Data Processing and Format Details: An illustration of how tabular medical records are converted into model training data: patient demographic labels, and clinical event sequences. The PyHealth implementation of `MedGenBoost` allows users to define arbitrary functions to handle code and label mapping. In the illustration, labels are encodings of age group, sex, and mortality status. Similarly, code mappings are a multi-hot vector representing the presence of clinical event codes in each visit, referred to as a "Patient Visit Vector". Patient Visits represent the ordered set of patient visit vectors.
